# Surface pre-reacted glass-ionomer eluate protects gingival epithelium from penetration by lipopolysaccharides and peptidoglycans via transcription factor EB pathway

**DOI:** 10.1371/journal.pone.0271192

**Published:** 2022-07-27

**Authors:** Hiroki Takeuchi, Yuta Kato, Naoko Sasaki, Keita Tanigaki, Shunsuke Yamaga, Ena Mita, Masae Kuboniwa, Michiya Matsusaki, Atsuo Amano

**Affiliations:** 1 Department of Preventive Dentistry, Graduate School of Dentistry, Osaka University, Suita, Osaka, Japan; 2 Joint Research Laboratory (TOPPAN) for Advanced Cell Regulatory Chemistry, Graduate School of Engineering, Osaka University, Suita, Osaka, Japan; 3 Department of Applied Chemistry, Graduate School of Engineering, Osaka University, Suita, Osaka, Japan; Lobachevsky University, RUSSIAN FEDERATION

## Abstract

Surface pre-reacted glass-ionomer (S-PRG) filler, produced by PRG technology for use with various dental materials, is bioactive and known to release ions from a glass-ionomer phase. We previously reported that coxsackievirus and adenovirus receptor (CXADR), a tight junction associated protein, was located in the epithelial barrier of gingival epithelium. In the present study, the tissue protective effects of an S-PRG eluate prepared with S-PRG filler were investigated using a three-dimensional human gingival epithelial tissue model. The results showed that the S-PRG eluate specifically induced CXADR expression at the transcriptional level of messenger RNA as well as the protein level, and also nuclear translocation of transcription factor EB (TFEB) in gingival epithelial cells. Furthermore, shigyakusan, a TFEB inhibitor, canceled induction of the CXADR protein by the S-PRG eluate. Additionally, gingival epithelial permeation by 40-kDa dextran, lipopolysaccharide, and peptidoglycan in the 3D-tissue models was prevented by the eluate, with those effects abrogated by knockdown of CXADR. These findings suggest that S-PRG eluate increases CXADR expression via the TFEB pathway, thus inhibiting penetration of bacterial virulence factors into subepithelial tissues.

## Introduction

Periodontal diseases are characterized by destruction of gingival tissues including alveolar bone, eventually leading to tooth exfoliation, with more than 796 million individuals worldwide estimated to be affected in a report presented in 2017 (GBD 2017 Disease and Injury Incidence and Prevalence Collaborators, 2017, Lancet) [1]. These diseases are initiated on epithelial surfaces of the subgingival compartment, while gingival epithelium functions as a physical innate barrier to protect microbial intrusion and also plays a central role in the innate immune system. The innate system is capable of precise recognition of microbial components, known as pathogen-associated molecular patterns (PAMPs). Those include lipopolysaccharides (LPS), which are endotoxins of gram-negative bacteria, and peptidoglycans (PGN), mesh-like patterns outside the plasma membrane of most bacteria, as prototypical classes of PAMPs that activate pro-inflammatory signaling pathways (Akira et al., 2006, Cell) [2].

Clinical periodontal treatment generally includes local or general administration of an antimicrobial agent to inhibit the harmful activities of periodontal pathogens. However, antimicrobial therapy may not be effective against drug-resistant bacteria, while it can also kill not only periodontal pathogens but good commensal bacteria as well (Ardila et al., 2010, J Periodontal Res) [3]. Hence, development of treatments that use factors related to establishment of a beneficial environmental for enhancing host defense against penetration by PAMPs into gingival epithelium is expected.

An alternative approach to protect gingival epithelium from bacterial pathogens is use of bioactive materials. By utilizing the reaction between fluoroboroaluminosilicate glass and a polyacrylic acid solution, pre-reacted glass-ionomer (PRG) technology has been applied to synthesize a surface-PRG (S-PRG) filler (Ikemura et al., 2003, Dent Mater) [4], which is currently used in various dental materials including composite resins, bonding agent cements, and resin sealants (Shimazu et al., 2012, J Clin Pediatr Dent; Ma et al., 2012, Dent Mater) [5, 6]. S-PRG filler releases six ions; fluoride (F^-^), sodium (Na^+^), borate (BO_3_^3-^), aluminum (Al^3+^), silicate (SiO_3_^2-^), and strontium (Sr^2+^). Silicate and fluoride are known to strongly induce remineralization of the dentin matrix (Saito et al., 2003, Caries res) [7], while strontium and fluoride improve the acid resistance of teeth by acting on hydroxyapatite to convert it to strontium-apatite (Featherstone et al., 1983, J Dent Res; Dedhiya et al., 1973, J Dent Res) [8, 9] and fluorapatite (Fetherstone et al., 1983, J Dent Res; Iijima et al., 1989, J Dent Res; Zero et al., 1988, J Dent Res) [8, 10, 11]. Although glass-ionomer cements are considered to be tissue compatible, the effects of ion components on barrier function in gingival epithelium have not been studied.

Epithelium in the area of the gingival sulcus is a stratified squamous tissue, in which gingival epithelial cells show cell-to-cell molecular adhesion and sealing complexes. In previous studies, we identified junctional adhesion molecule 1 (JAM1), as well as coxsackievirus and adenovirus receptor (CXADR) as barrier proteins in gingival epithelium that protect against bacterial pathogens (Takeuchi et al., 2019, PLoS Pathog; 2021, Cell Microbiol) [12, 13]. In nonpolarized cells, CXADR has been shown to localize to homotypic intercellular contacts and function as a barrier against paracellular solute movement (Cohen et al., 2001, Proc Natl Acad Sci USA) [14]. Eukaryotic cells are composed of interior compartments that are themselves enclosed by lipid membranes. Extracellular substrates are transported to endocytic pathways including lysosomes, while lysosomes function to break down large molecules, such as proteins, lipids, nucleic acids, and carbohydrates (Ballabio et al., 2019, Nat Rev Mol Cell Biol) [15]. Furthermore, they have also been reported to function as sensors for lysosomal Ca^2+^, thus promoting nuclear translocation of the transcription factor EB (TFEB) to regulate autophagy (Medina et al., 2015, Nat Cell Biol) [16]. Hence, the six ions present in S-PRG eluate may have effects on host transcriptional levels, including JAM1 and CXADR.

This study was conducted to examine the effects of S-PRG eluate prepared with an S-PRG filler on gingival epithelial barrier function using three-dimensional (3D) multilayered tissue models. The results showed that S-PRG eluate induced gene and protein expression of CXADR, which was found to be inhibited by shigyakusan, a TFEB inhibitor. Thus, gingival epithelial impermeability to LPS and PGN was induced in the 3D tissue models, leading to a stronger gingival epithelial barrier. These findings suggest that S-PRG eluate is a bioactive material with potential for use to protect against initiation of periodontal diseases.

## Results

### S-PRG eluate induced CXADR expression by gingival epithelial cells

**Fig 1:**
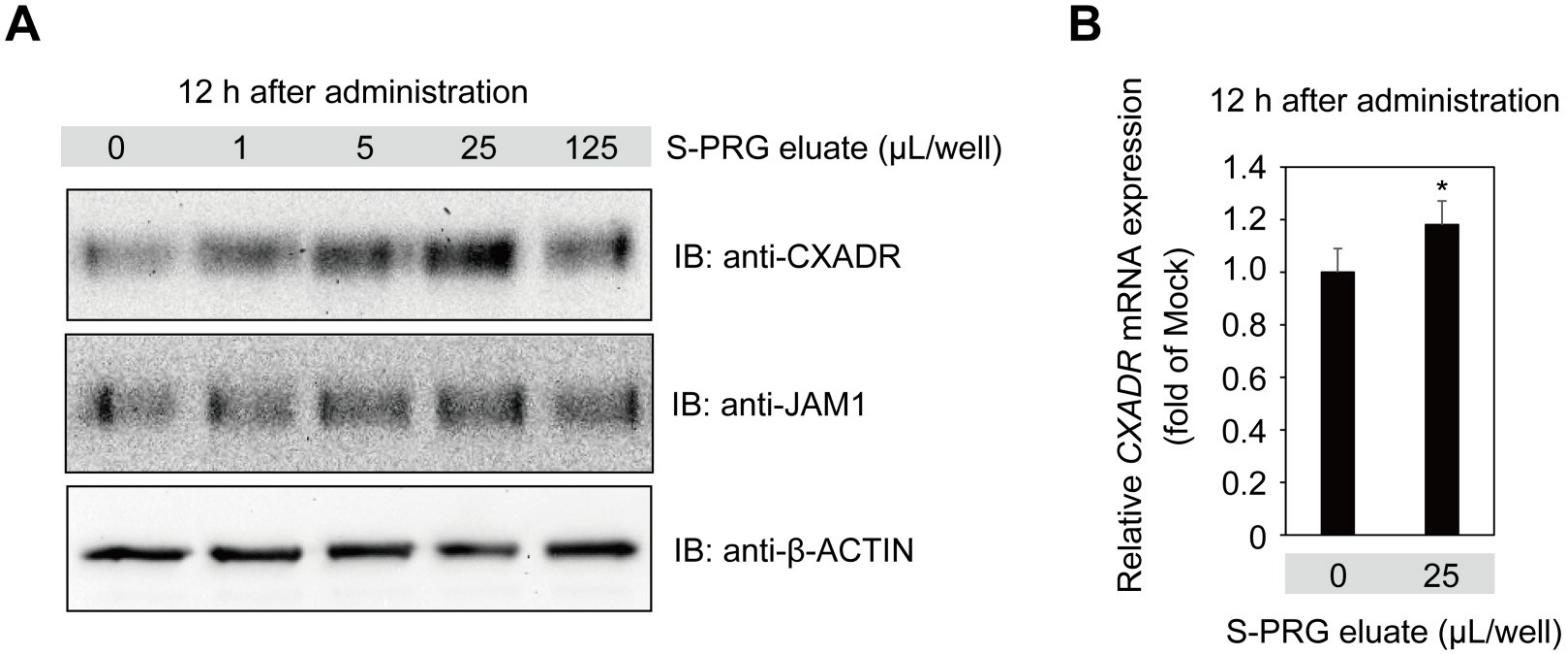
S-PRG eluate induces CXADR expression in IHGE cells. **(A)** IHGE cells in six-well plates were treated with S-PRG eluate for 12 hours, then analyzed by immunoblotting with the indicated antibodies. β-ACTIN was used as a loading control. IB, immunoblot. **(B)** Effects of *CXADR* mRNA expression in IHGE cells in six-well plates treated with S-PRG eluate for 12 hours. Results are expressed as fold change relative to Mock (no S-PRG eluate) and are presented as the mean of six technical replicates. *p<0.05. Results shown are representative of two biological replicates. See also S1 Fig.

Calcium ion has been shown to be one of the key factors for assembly and sealing of tight junctions in Madin-Darby canine kidney (MDCK) cells (Cereijido M, et al., 1978, J Cell Biol; Martinez-Palomo et al., 1980, J Cell Biol) [17, 18], while S-PRG eluate is known to release several types of ions, including Al, B, Na, Si, Sr, and F (Fujimoto et al., 2010, Dent Mater) [19]. Those findings prompted us to examine the effects of S-PRG eluate on gingival epithelial barrier function. To determine whether the six ions of S-PRG eluate are transferred to gingival epithelium, immortalized human gingival epithelial (IHGE) cells were treated with S-PRG eluate for one hour, after which the level of each of the ions was found to be increased in culture media (S1A Table). Furthermore, it was shown that BO_3_^3-^, Sr^2+^, Na^+^, and F^-^ ions were increased in IHGE cells (S1B Table). These results suggest that ions of S-PRG eluate are taken in by gingival epithelial cells. To determine the effects to induce expression of CXADR and/or JAM1 at the endogenous protein level, IHGE cells were next treated with S-PRG eluate for 12 hours. Immunoblot findings revealed increased levels of CXADR in IHGE cells induced by S-PRG eluate at a volume of 25 μL, whereas there were negligible effects on JAM1 (Fig 1A). Furthermore, quantitative real-time PCR showed that the level of CXADR mRNA in IHGE cells was increased by administration of S-PRG eluate (Fig 1B). Notably, mRNA levels of CLAUDIN1 (CLDN1) and OCCLUDIN (OCLN), major tight junction transmembrane proteins (Tsukita et al., 2001, Nat Rev Mol Cell Biol) [20], were not significantly changed (S1 Fig). These results indicated that the S-PRG eluate specifically enhanced CXADR expression.

**Fig 2:**
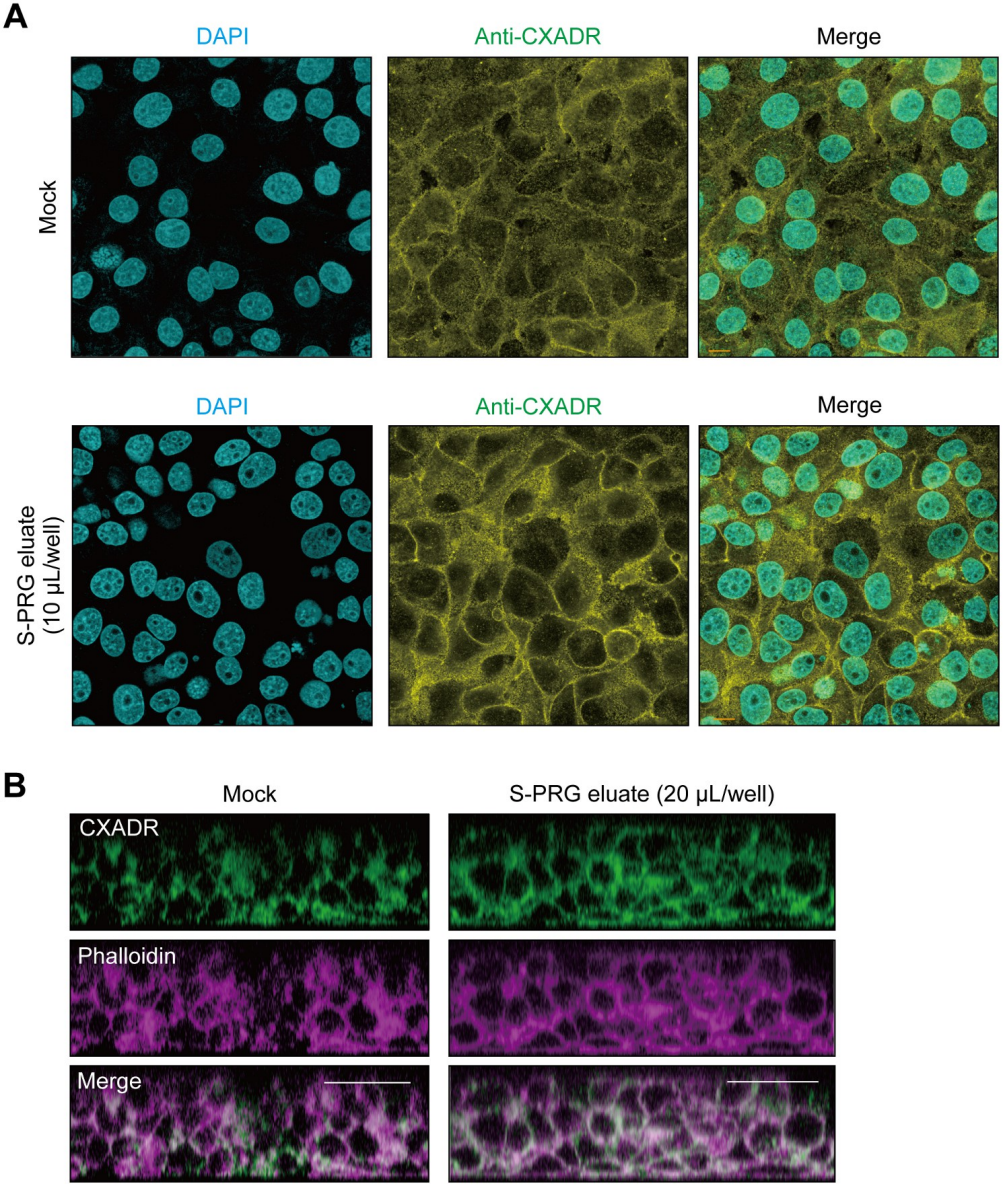
Confocal microscopic images of CXADR in gingival epithelial tissues treated with S-PRG eluate. **(A)** IHGE cells in 12-well plates were treated with S-PRG eluate for 12 hours, then fixed, stained with DAPI (cyan) and anti-CXADR (yellow), and analyzed by confocal microscopy. Scale bars, 10 μm. **(B)** Gingival epithelial tissues on coverslips in 24-well plates were treated with S-PRG eluate for 12 hours, then fixed, stained with anti-CXADR (green) and Alexa Fluor 633–conjugated phalloidin (magenta), and analyzed by confocal microscopy. Scale bars, 30 μm. Results shown are representative of two biological replicates. See also S2 Fig.

The effects of S-PRG eluate on localization of CXADR in IHGE cells was also examined. CXADR signal intensity was found to be increased in the peripheral area of IHGE cells treated with S-PRG eluate for 12 hours (Fig 2A). To assess the contribution of S-PRG to CXADR expression deeper in epithelium tissues, 3D-tissue models of IHGE cells generated by a cell-accumulation technique were used (Nishiguchi et al., 2011, Adv Mater) [21] (S2 Fig). Examinations showed that S-PRG eluate increased CXADR at 12 hours after administration, even in tissues 3–4 layers below the surface (Fig 2B). Thus, it was indicated that S-PRG eluate increases CXADR expression even in deeper layers of human gingival epithelial tissues.

### TFEB pathway involved in CXADR expression induced by S-PRG eluate

**Fig 3:**
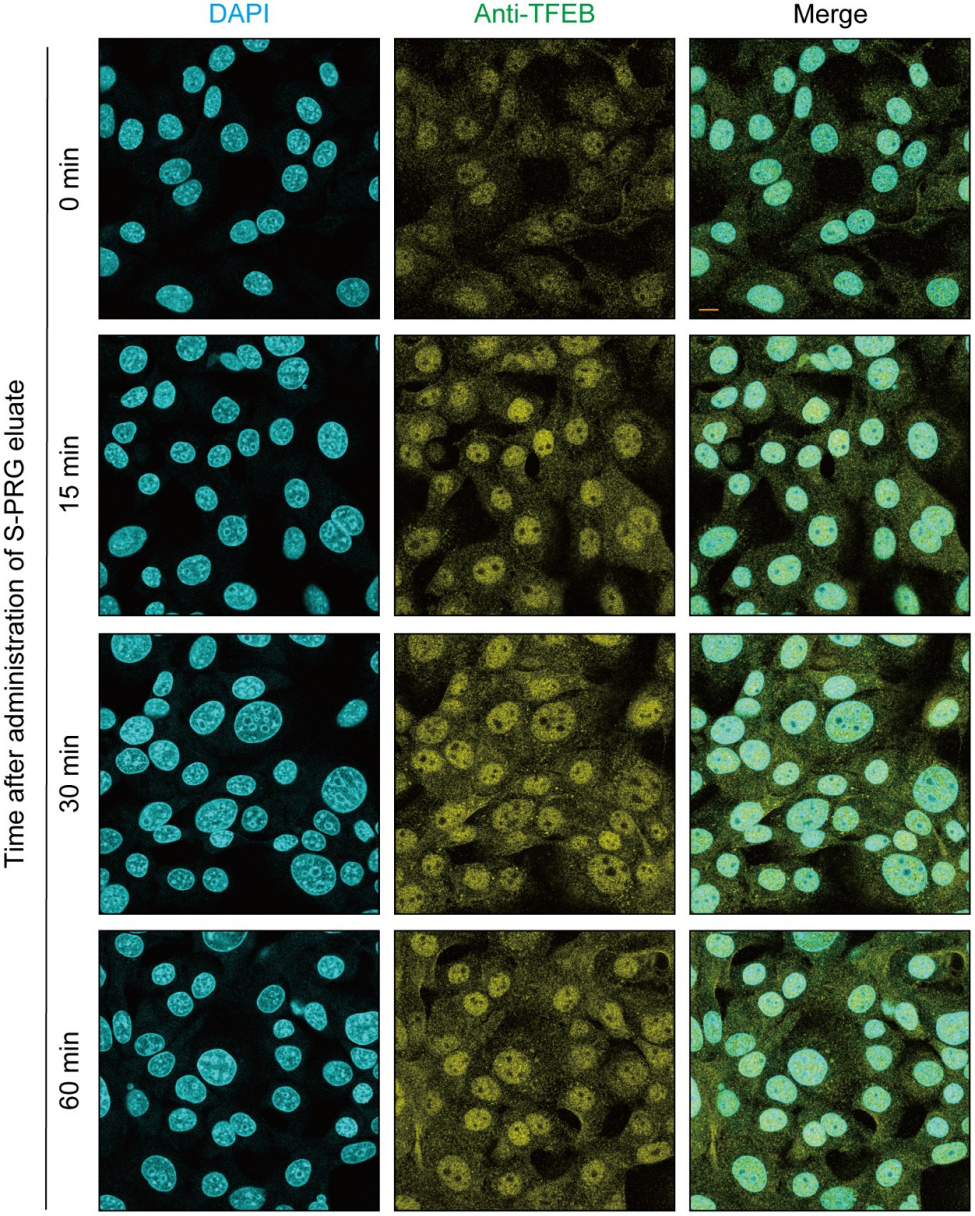
S-PRG increases nuclear translocation of TFEB in IHGE cells. IHGE cells in 12-well plates were treated with 10 μL of S-PRG eluate for the indicated time periods or left unstimulated, then fixed, stained with DAPI (cyan) and anti-TFEB (yellow), and analyzed by confocal microscopy. Scale bars, 10 μm. Results shown are representative of two biological replicates.

The transcription factor involved in CXADR expression in gingival epithelial cells was examined after stimulation with S-PRG eluate. TFEB, a master gene for lysosomal biogenesis (Sardiello et al., 2009, Science) [22], regulates expression of lysosomal hydrolases and membrane proteins, which are involved in autophagy (Settembre et al., 2011 Science) [23]. With nutrient depletion and an aberrant lysosomal storage condition, TFEB is translocated from the cytoplasm to the nucleus, leading to activation of target genes. Hence, it is possible that S-PRG eluate is sensed by the TFEB pathway, which could lead to further expression of CXADR. As shown in Fig 3, S-PRG eluate clearly induced nuclear translocation of TFEB at 30 minutes after administration, indicating that it activated the TFEB pathway in the gingival epithelial cells.

Other transcriptional factors were also investigated. In the canonical pathway, stimuli such as bacterial products and proinflammatory cytokines activate nuclear factor-κB (NF-κB) and activator protein-1 (AP-1). NF-κB is comprised of homo- and hetero-dimers containing combinations of RelA/p65 subunits (Zhang et al., 2017, Cell) [24], while AP-1 is a heterodimer composed of proteins belonging to c-Fos and c-Jun (Chiu et al., 1988, Cell) [25]. We examined whether S-PRG eluate also induces NF-κB p65 and the c-JUN/c-FOS pathway. As shown in S3–S5 Figs, S-PRG eluate did not induce nuclear translocation of NF-κB p65, c-FOS, or c-JUN from cytosol in IHGE cells, thus suggesting that it specifically activates the TFEB pathway in gingival epithelial cells.

**Fig 4:**
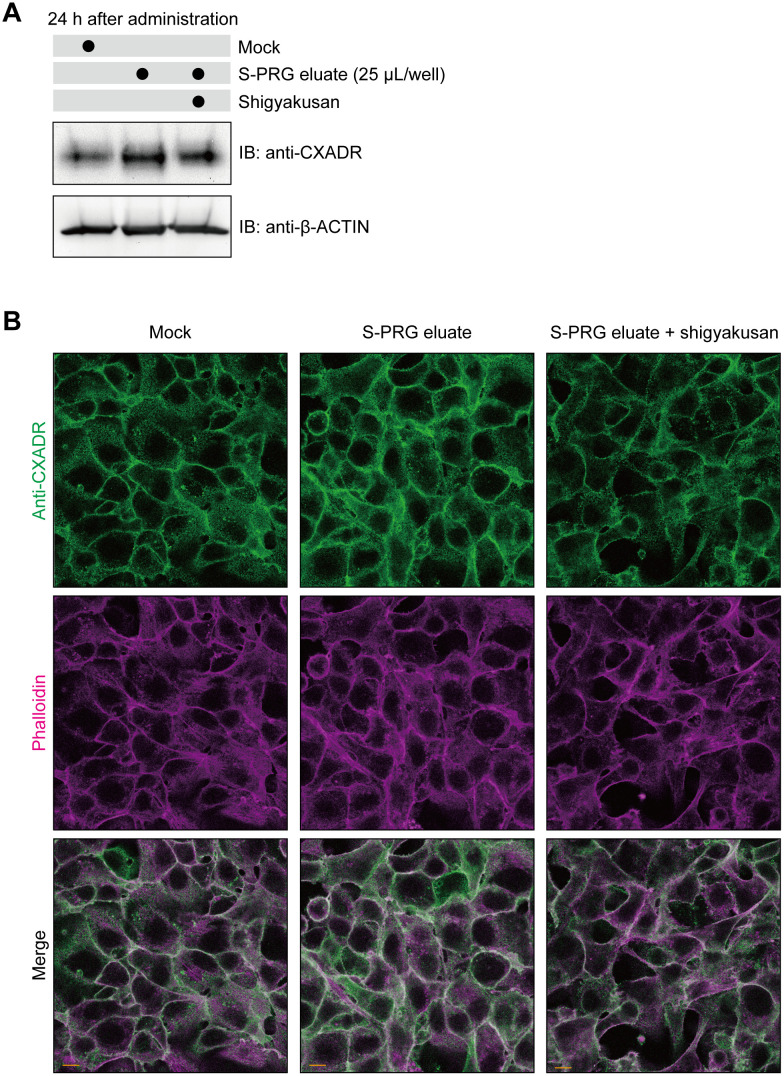
Shigyakusan disturbs S-PRG eluate-induced CXADR expression in IHGE cells. **(A)** IHGE cells in six-well plates were treated with S-PRG eluate with or without shigyakusan for 24 hours, or left unstimulated, then analyzed by immunoblotting with the indicated antibodies. β-ACTIN was used as a loading control. **(B)** IHGE cells in 12-well plates were treated with 10 μL of S-PRG eluate with or without shigyakusan for 24 hours, or left unstimulated, then fixed, stained with anti-CXADR (green) and Alexa Fluor 633-conjugated phalloidin (magenta), and analyzed by confocal microscopy. Scale bars, 10 μm. Results shown are representative of two biological replicates.

Shigyakusan is comprised of bupleurum root, peony root, immature orange, and glycyrrhiza boiled together, and has been reported to inhibit nuclear translocation of TFEB (Ikari et al., 2020, PLoS One) [26]. To examine its effects, IHGE cells were treated with S-PRG eluate, and with or without this TFEB inhibitor. The results showed that shigyakusan effectively disrupted TFEB nuclear translocation even after stimulation with S-PRG eluate (S6 Fig), while CXADR expression induced by the eluate was also blocked by shigyakusan (Fig 4A). As for localization of CXADR in gingival epithelial cells, shigyakusan blocked its expression induced on cell surfaces (Fig 4B). Together, these results suggest that the TFEB pathway promotes CXADR expression induced by S-PRG eluate.

### S-PRG eluate prevents permeation to LPS and PGN in gingival epithelium

**Fig 5:**
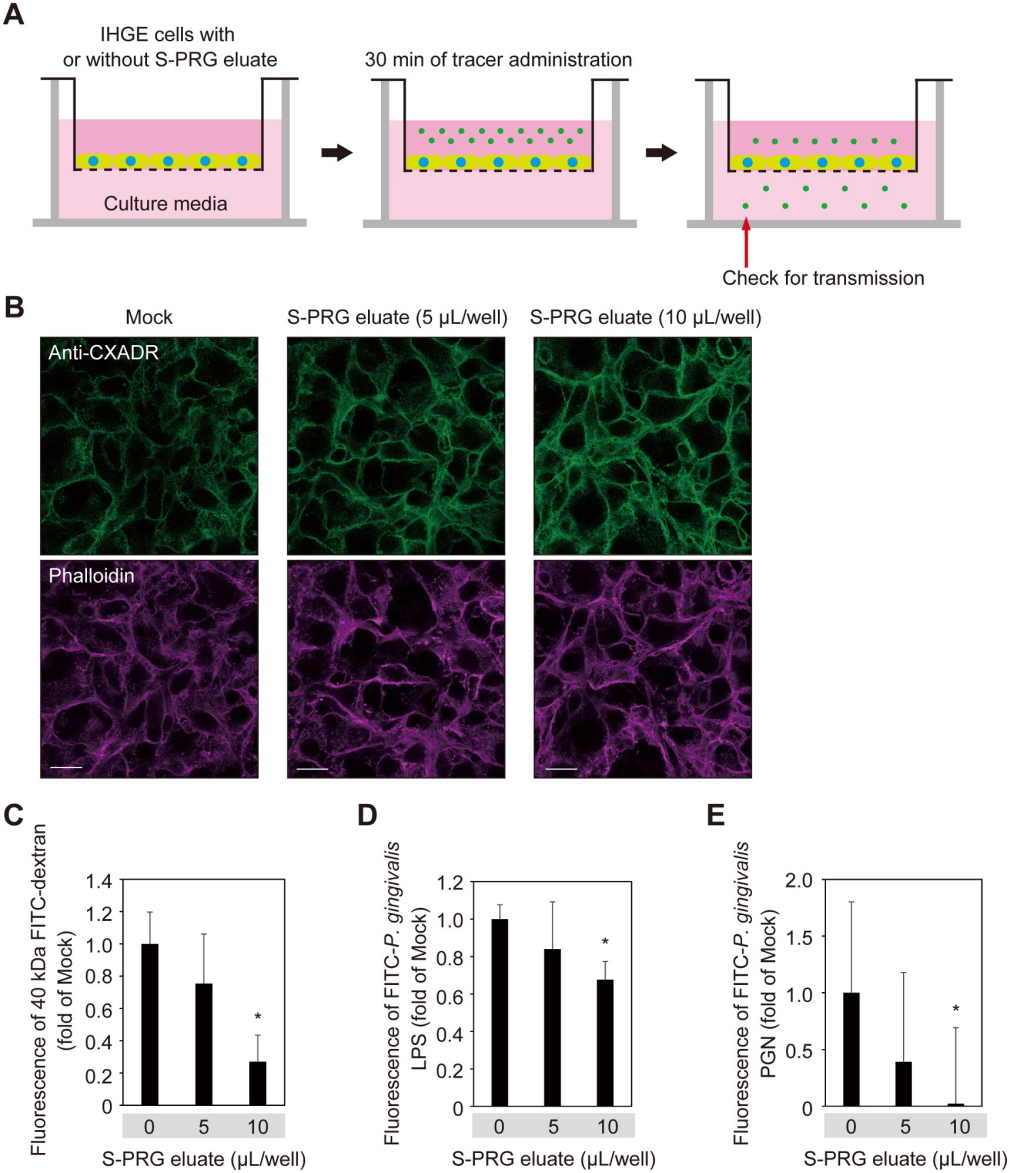
S-PRG eluate prevents permeation of LPS and PGN in IHGE cells. **(A)** Schematic image of culture insert system. Monolayers of IHGE cells were cultured in culture inserts in 12-well plates. Following 12 hours of incubation with S-PRG eluate, an FITC-labeled tracer was added to the culture medium in the upper compartment. After an additional 30 minutes of incubation, transmission of the tracer from the upper to lower compartment was analyzed by spectrometry. **(B)** IHGE cells in 12-well plates were treated with S-PRG eluate for 12 hours, then fixed, stained with anti-CXADR (green) and Alexa Fluor 633–conjugated phalloidin (magenta), and analyzed by confocal microscopy. Scale bars, 20 μm. **(C-E)** Gingival epithelial cell permeability to FITC-labeled dextran (40 kDa) (C), *P*. *gingivalis* LPS (D), or PGN (E) after treatment with or without S-PRG eluate. Results are expressed as fold change relative to Mock (no S-PRG eluate) and are shown as the mean ± SD of eight technical replicates. *p<0.05, Dunnett’s test. Results shown are representative of two biological replicates.

**Fig 6:**
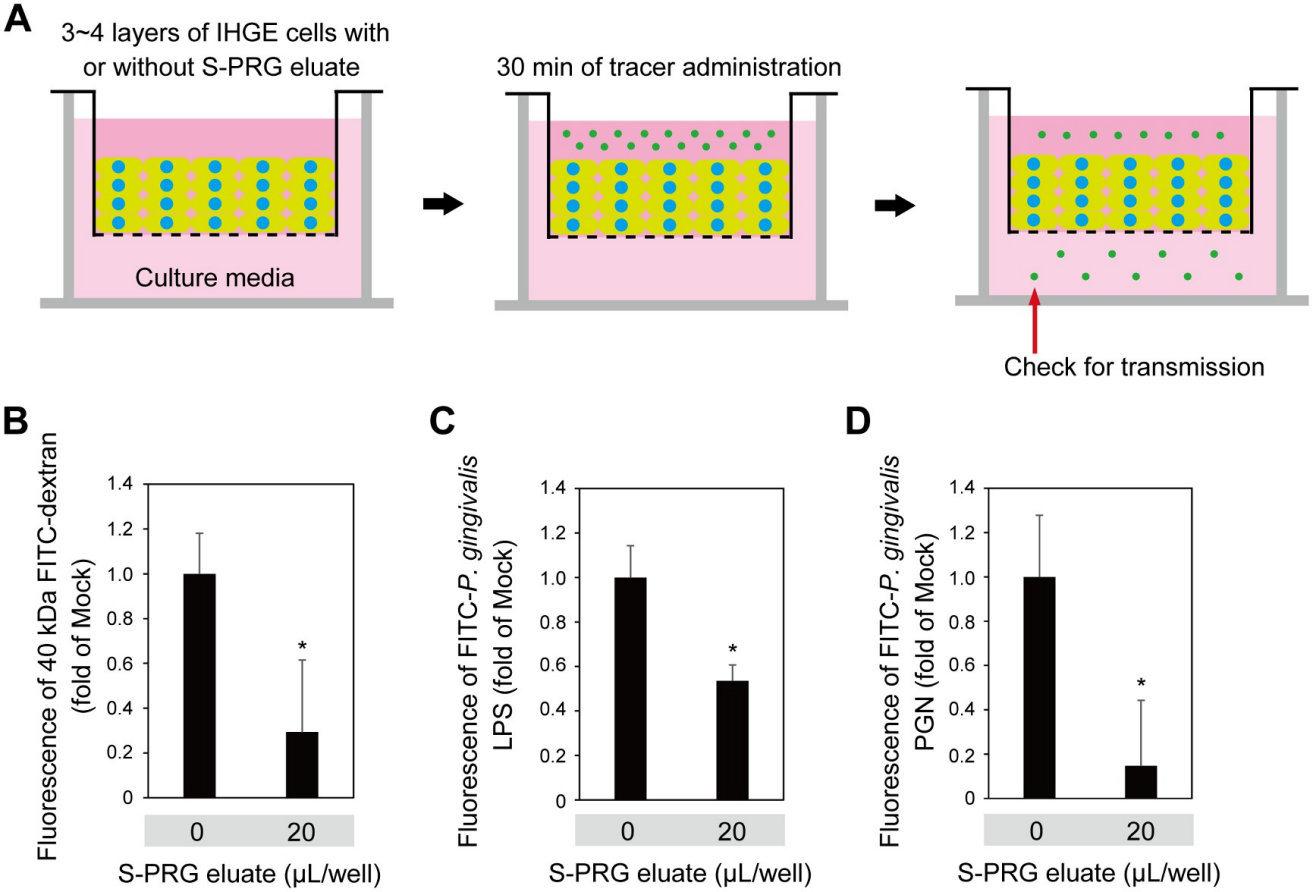
S-PRG eluate prevents permeation of LPS and PGN in gingival epithelial tissues. **(A)** Schematic image of culture insert system. Gingival epithelial tissues were cultured in culture inserts in 12-well plates. Following 12 hours of incubation with S-PRG eluate, an FITC-labeled tracer was added to the culture medium in the upper compartment. After an additional 30 minutes of incubation, transmission of the tracer from the upper to lower compartment was analyzed by spectrometry. **(B-D)** Permeability of FITC-labeled dextran (40 kDa) (B), *P*. *gingivalis* LPS (C), or PGN (D) in gingival epithelial tissues treated with or without S-PRG eluate. Results are expressed as fold change relative to Mock (no S-PRG eluate) and shown as the mean ± SD of eight technical replicates. *p<0.05. Results shown are representative of two biological replicates.

To assess the contribution of S-PRG eluate to gingival epithelium permeability, assays using a small-molecule fluorescent probe were performed (Fig 5A). Following treatment with S-PRG eluate, IHGE monolayers showed impermeability when exposed to 40-kDa FITC-dextran, FITC-labeled *P*. *gingivalis* LPS, and FITC-labelled *P*. *gingivalis* PGN, with the effect increased in a dose-dependent manner (Fig 5B–5E). Additionally, permeability assays were performed using 3D-tissue models (Fig 6A), which also indicated increased tissue impermeability induced by S-PRG eluate toward 40-kDa FITC-dextran, FITC-labeled *P*. *gingivalis* LPS, and FITC-labelled *P*. *gingivalis* PGN (Fig 6B–6D). Thus, it was concluded that S-PRG eluate increases the impermeability of human gingival epithelium toward LPS and PGN.

### S-PRG eluate enhances barrier function of gingival epithelium in CXADR-dependent manner

**Fig 7:**
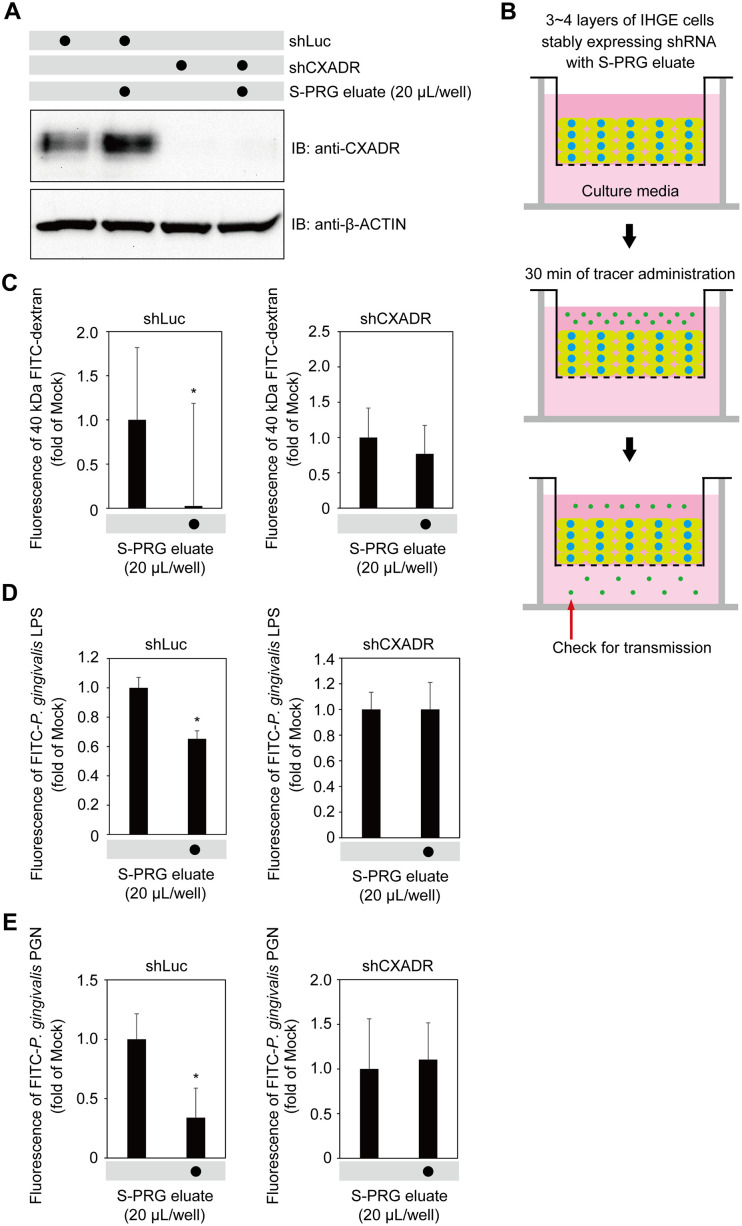
S-PRG eluate induces CXADR expression in gingival epithelium, preventing penetration by LPS and PGN. **(A)** IHGE cells stably expressing shLuc or shCXADR in six-well plates were treated with S-PRG eluate for 12 hours, then analyzed by immunoblotting with the indicated antibodies. **(B)** Schematic image of culture insert system. Gingival epithelial tissues stably expressing shLuc or shCXADR were cultured in culture inserts. Following 12 hours of incubation with S-PRG eluate, an FITC-labeled tracer was added to the culture medium in the upper compartment. After an additional 30 minutes of incubation, transmission of the tracer from the upper to lower compartment was analyzed by spectrometry. See also S5 Fig. **(C-E)** Permeability of gingival epithelial tissues to FITC-labeled dextran (40 kDa) (C), *P*. *gingivalis* LPS (D), or PGN (E) in 12-well plates expressing indicated shRNA, with or without S-PRG eluate. Results are expressed as fold change relative to Mock (no S-PRG eluate) and shown as the mean ± SD of eight technical replicates. *p<0.05. Results shown are representative of two biological replicates.

To assess the influence of CXADR on the effects of S-PRG eluate, IHGE cells expressing short hairpin RNA against CXADR (shCXADR) were generated to block CXADR expression. Immunoblot findings of the level of expression in IHGE cells expressing shCXADR with or without S-PRG eluate confirmed that CXADR expression induced by S-PRG eluate was sufficiently abrogated by shCXADR knockdown (Fig 7A). Next, 3D-tissue models of IHGE cells expressing shCXADR were generated and CXADR localization in gingival epithelial tissues was analyzed. As shown in S7 Fig, shCXADR caused CXADR to disappear even in tissues 3–4 layers below the surface. Permeability assays of gingival epithelial tissues expressing shCXADR with exposure to S-PRG eluate were then performed (Fig 7B), which indicated decreased permeation of 40-kDa dextran, *P*. *gingivalis* LPS, and *P*. *gingivalis* PGN in tissues expressing short hairpin RNA against *Renilla* luciferase (shLuc), used as a control, while that decrease was abrogated by CXADR depletion. Based on these results, it was concluded that the effects of S-PRG eluate on human gingival epithelium tissue permeability is dependent on CXADR.

## Discussion

This is the first known report to present results demonstrating the effects of S-PRG eluate released from dental cement on gingival epithelium. Glass-ionomer cement and composite resin are widely used when setting a prosthetic appliance onto teeth, as well as to fill tooth cavities as part of dental treatment. Thus, research exploring the biochemical effects of dental cements have mainly focused on hard tissues related to prevention of dental caries, but rarely on soft tissues. When an S-PRG filler that contains cement or resin is placed so as to face gingival epithelium, the S-RPG eluate subsequently released potentially protects gingival tissues from PAMP penetration. Since S-PRG filler has an ability to recharge six ions, prolonged release of S-PRG eluate for a longer time period can be expected, which then functions as a stabilizer of gingival heath.

Intracellular calcium has been reported to be crucial for tight junction integrity (Ye et al., 1999, Am J Physiol) [27]. Lipid bilayers show negligible transmission of ions into host cells. In contrast with S-PRG eluate, several ions are likely internalized by ion channels to lysosomes, leading to an enhanced CXADR-derived barrier function in gingival epithelial cells. It was previously reported that calcium efflux from lysosomes induced TFEB nuclear translocation in an autophagy-related gene-dependent manner (Nakamura et al., 2020, Nat Cell Biol) [28]. In the present study, TFEB inhibitors were shown to abrogate CXADR expression induced by S-PRG eluate (Fig 4A and 4B). A future study to examine possible crosstalk between internalized ions of S-PRG eluate and the TFEB/autophagic pathway would be of interest.

A basal level of NF-κB activation is necessary for cellular homeostasis at the intestinal epithelial interface (Wullaert et al., 2011, Cell Res) [29]. In contrast, persistently elevated NF-κB and AP-1 activation causes induction of expression of proinflammatory chemokines and cytokines by host cells, leading to inflammation. Hence, in regard to gingival epithelium barrier integrity, we consider that S-PRG eluate has an advantage, as it does not change the NF-κB and AP-1 pathways from their basal level. Other studies have shown that commensal bacteria contribute to fortification of the intestinal epithelial barrier by promoting epithelial cell maturation and angiogenesis (Hooper et al., 2001, Science; Stappenbeck et al., 2002, Proc Natl Acad Sci USA) [30, 31]. Indeed, we also previously found that *Streptococcus gordonii*, a commensal bacterium, had negligible effects on JAM1 and CXADR expression levels in IHGE cells (Takeuchi et al., 2019, PLoS Pathog; 2021 Cell Microbiol) [12, 13]. Therefore, when exploring bioactive materials, the finely balanced activity of transcription factors with barrier proteins is key for prevention of periodontitis and S-RPG eluate likely meets the criteria.

For research related to drug development, *in vitro* methods are considered to be an acceptable alternative to animal experiments from the viewpoint of the three Rs; replacement, reduction, and refinement (Russel & Burch, 1954; Smith, 1978) [32, 33]. Along this line, the 3D-tissue models of gingival epithelium used in the present study are considered to be potentially beneficial for confirming the effects of a new material, such as S-PRG eluate. Additionally, the results illustrate the molecular basis of the target barrier protein and its transcriptional factor utilized by S-PRG eluate shown by use of a 3D-model, factors that have been rarely examined using native human tissues.

There is considerable interest regarding the interactions between periodontal and systemic diseases, and it is also likely that maintenance of a good oral condition is linked to better overall health. Periodontal bacteria and host defense factors must remain in balance to maintain oral health, thus it is necessary to protect the epithelial barrier from bacterial virulence factors. Hence, it will be important to discover additional effects of S-PRG eluate on gingival epithelium tissues as well as the epithelial barrier.

## Materials and methods

### Cell culture

IHGE cells (kindly provided by Shinya Murakami, Osaka University) were maintained in Humedia KG-2 (Kurabo), as previously described (Murakami et al., 2002, J Dent Res) [34]. Cell and three-dimensional cultures were performed as previously described (Takeuchi et al., 2019, PLoS Pathog) [12]. S-PRG eluate was diluted with IHGE cell culture medium, as indicated in the description of each experiment.

### S-PRG eluate preparation

S-PRG eluate was prepared as previously described (Fujimoto et al., 2010, Dent Mater) [19] and provided by Shofu Inc. (Kyoto, Japan). Briefly, S-PRG filler was gently mixed with an equal amount of distilled water at room temperature for 24 hours, followed by centrifugation at 3000 x *g* at 23°C for six hours to separate filler from liquid. The supernatant was then filtered (pore size 0.45 μm) to remove any residual insoluble material and the resulting filtrate was used as S-PRG eluate. The concentrations of Al^3+^, BO_3_^3-^, Na^+^, SiO_3_^2-^, and Sr^2+^ ions released from the S-PRG filler were determined using an emission spectrophotometer (ICPS-8000, Shimazu), while that of F^-^ was examined with an F^-^ electrode (Model 9609BNWP, Orion Research) using an ion selective electrode meter (Model 720A, Orion Research). Ion concentrations in the S-PRG eluate were as follows: Al^3+^ = 0.52 nmol/L, BO_3_^3-^ = 132.60 nmol/L, Na^+^ = 19.58 nmol/L, SiO_3_^2-^ = 0.49 nmol/L, Sr^2+^ = 1.64 nmol/L, and F^-^ = 6.50 nmol/L.

### Shigyakusan preparation

Shigyakusan was obtained from Kyushin Kampo, Tokyo, Japan, and prepared as previously described (Ikari et al., 2021, PLoS One) [26]. Ingredients comprising shigyakusan products are available at STORK (http://mpdb.nibiohn.go.jp/stork/), which is maintained by the National Institute of Health Sciences (NIHS) of Japan. Shigyakusan extract was soaked in water at 40 mg/mL in a 1.5-mL tube and incubated at 98°C for 5 minutes. Debris was sedimented by centrifugation at 20,400 x *g* for 10 minutes, then supernatants were aliquoted and stored at -20°C. Shigyakusan extract (final concentration 400 μg/mL) was added to IHGE cell culture media, then incubation was performed as indicated in Figs 3 and 4, and S4 Fig.

### Antibodies and reagents

The antibodies and reagents used in this study are shown in S2 Table.

### Immunoblotting

Immunoblotting was performed as previously described (Takeuchi et al., 2019, PLoS Pathog) [12]. Immunoreactive bands were detected using Pierce ECL Western Blotting Substrate (Thermo Scientific) and ChemiDoc XRS (Bio-Rad). Images were acquired using the Quantity One software package (Bio-Rad).

### Immunocytochemistry

Immunostaining was performed as previously described (Takeuchi et al., 2019, PLoS Pathog) [12]. Images were acquired with a confocal laser microscope (TCS SP8; Leica Microsystems) using a 64× oil-immersion object lens with a numerical aperture of 1.4. Acquired images were analyzed using the Application Suite X software package (Leica Microsystems).

### Reverse transcription PCR

Total RNA was extracted from IHGE cells using TRIzol (Thermo Fisher Scientific). Complementary DNA was synthesized using an iScript cDNA Synthesis Kit (Bio-Rad). Reverse transcription PCR was performed with primers, as follows: CXADR forward, 5’-ATGCCCACTTCATGGTTAGC-3’; reverse, 5’-GCGCTAGAGCAAGCAAAGTT-3’, CLDN1 forward, 5’- CCGTTGGCATGAAGTGTATG-3’; reverse, 5’-CCAGTGAAGAGAGCCTGACC-3’, and OCLN forward, 5’-TCCAATGGCAAAGTGAATGA-3’; reverse, 5’- GCAGGTGCTCTTTTTGAAGG-3’, and GAPDH forward, 5’-CCACCCATGGCAAATTCCATGGCA-3’; reverse, 5’-TCTAGACGGCAGGTCAGGTCCACC-3’. Real-time PCR was performed using a Rotor Gene Q (Qiagen) with a KAPA SYBR FAST Universal qPCR Kit (KAPA Biosystems). The amplicon level in each sample was normalized against the corresponding level of GAPDH mRNA content using the 2–ΔΔCt method.

### RNA interference

A plasmid encoding shRNA was constructed by ligation of linear DNA (Sigma-Aldrich) into pSIREN-RetroQ (631526, Clontech). pSIREN-RetroQ-shCXADR #317 plasmid (target sequences 5’-ATGTAACGAATTTACAACTGTCA-3’) was used for generation of the siRNA duplex in cells (Takeuchi et al., 2021, Cell Microbiol) [13]. pSIREN-RetroQ-shLuc plasmid was produced as previously described (Takeuchi et al., 2019, PLoS Pathog) [12]. IHGE cells were transfected with shRNA-encoding plasmid using FuGENE 6 (Promega). Seventy-two hours after transfection, cells stably expressing shRNA were selected with use of puromycin (2 μg mL^-1^; Invitrogen).

### Epithelial barrier function assay

Preparation of an FITC-labeled tracer and epithelial barrier function assays were performed as previously described (Takeuchi et al., 2019, PLoS Pathog) [12]. Fluorescence intensity was determined using a 1420 ARVO *X* device (PerkinElmer). Data were acquired using the WorkOut Plus software package (PerkinElmer).

### Ion concentration determination in IHGE cells and culture media

IHGE cells in 10-cm tissue culture dishes were treated with S-PRG eluate. After one hour of incubation, culture medium was discarded and cells were collected in 10 mL of distilled water. The cells were then mechanically disrupted by vigorous passage through a 21-gauge needle eight times on ice. Concentrations of the Al^3+^, BO_3_^3-^, Na^+^, SiO_3_^2-^, and Sr^2+^ ions in cytoreductive solutions (10 ml of distilled water/dish) and culture media (10 mL of Humedia KG2/dish) were determined using inductively coupled plasma atomic emission spectroscopy (ICP-AES; ICPS-8000, Shimadzu), while that of F^-^ was examined with an F^-^ electrode (Model 9609BNWP, Orion Research) using an ion selective electrode meter (Model 720A, Orion Research).

### Statistical analysis

P values were determined using a two-tailed *t* test and Dunnett’s test with the Excel software package (Microsoft), with p<0.05 considered to indicate significance.

## Supporting information

S1 Fig S-PRG eluate does not increase CLDN1 or OCLN expression in IHGE cells. **(A, B)** Effects of *CLDN1* (A) and *OCLN* (B) mRNA expression in IHGE cells in six-well plates treated with S-PRG eluate for 12 hours. Results are expressed as fold change relative to Mock (no S-PRG eluate) and presented as the mean of five technical replicates. Results shown are representative of two biological replicates.
(TIF)

S2 Fig Confocal microscopic images of gingival epithelial tissues treated with S-PRG eluate. Gingival epithelial tissues on coverslips in 24-well plates were treated with S-PRG eluate for 12 hours, then fixed, stained with DAPI (cyan) and anti-CXADR (gray), and analyzed by confocal microscopy. Scale bars, 30 μm.
(TIF)

S3 Fig S-PRG eluate does not induce nuclear translocation of NF-κB in IHGE cells. IHGE cells in 12-well plates were treated with 10 μL of S-PRG eluate for the indicated time periods or left unstimulated, then fixed, stained with DAPI (cyan) and anti-NF-κB (yellow), and analyzed by confocal microscopy. Scale bars, 10 μm. Results shown are representative of two biological replicates.
(TIF)

S4 Fig S-PRG eluate does not induce nuclear translocation of c-FOS in IHGE cells. IHGE cells in 12-well plates were treated with 10 μL of S-PRG eluate for the indicated time periods or left unstimulated, then fixed, stained with DAPI (cyan) and anti-c-FOS (yellow), and analyzed by confocal microscopy. Scale bars, 10 μm. Results shown are representative of two biological replicates.
(TIF)

S5 Fig S-PRG eluate does not induce nuclear translocation of c-JUN in IHGE cells. IHGE cells in 12-well plates were treated with 10 μL of S-PRG eluate for the indicated time periods or left unstimulated, then fixed, stained with DAPI (cyan) and anti-c-JUN (yellow), and analyzed by confocal microscopy. Scale bars, 10 μm. Results shown are representative of two biological replicates.
(TIF)

S6 Fig Shigyakusan disturbs nuclear translocation of TFEB induced by S-PEG eluate. IHGE cells in 12-well plates were treated with 10 μL of S-PRG eluate with or without shigyakusan for the indicted time periods, or left unstimulated, then fixed, stained with DAPI (cyan) and anti-TFEB (yellow), and analyzed by confocal microscopy. Scale bars, 10 μm. Results shown are representative of two biological replicates.
(TIF)

S7 Fig Confocal microscopy images of gingival epithelial tissues expressing shLuc or shCXADR. Gingival epithelial tissues stably expressing shLuc or shCXADR on coverslips in 24-well plates were fixed, stained with anti-CXADR (green) and Alexa Fluor 568-conjugated phalloidin (magenta), and analyzed by confocal microscopy. Scale bars, 30 μm.
(TIF)

S8 Fig Immunoblots performed in this study. (PDF)

S1 Table Ion concentration in cytoreductive solution for IHGE cell culture media. IHGE cells in a 10-cm tissue culture dish were treated with S-PRG eluate. After one hour of incubation, cells and culture media were collected and analyzed by ICP-AES for Al^3+^, BO_3_^3-^, Na^+^, SiO_3_^2-^, and Sr^2+^, or an ion selective electrode meter for F^-^. Results are expressed in parts per million (ppm) and presented as the mean of five technical replicates. *p<0.05.
(TIF)

S2 Table Antibodies and reagents used in this study. (TIF)
