# Supplementary figures and images for: Surface pre-reacted glass-ionomer eluate protects gingival epithelium from penetration by lipopolysaccharides and peptidoglycans via transcription factor EB pathway
Source: PLoS One. 2022 Jul 27;17(7):e0271192. doi: 10.1371/journal.pone.0271192 (PMC9328573; doi:10.1371/journal.pone.0271192)

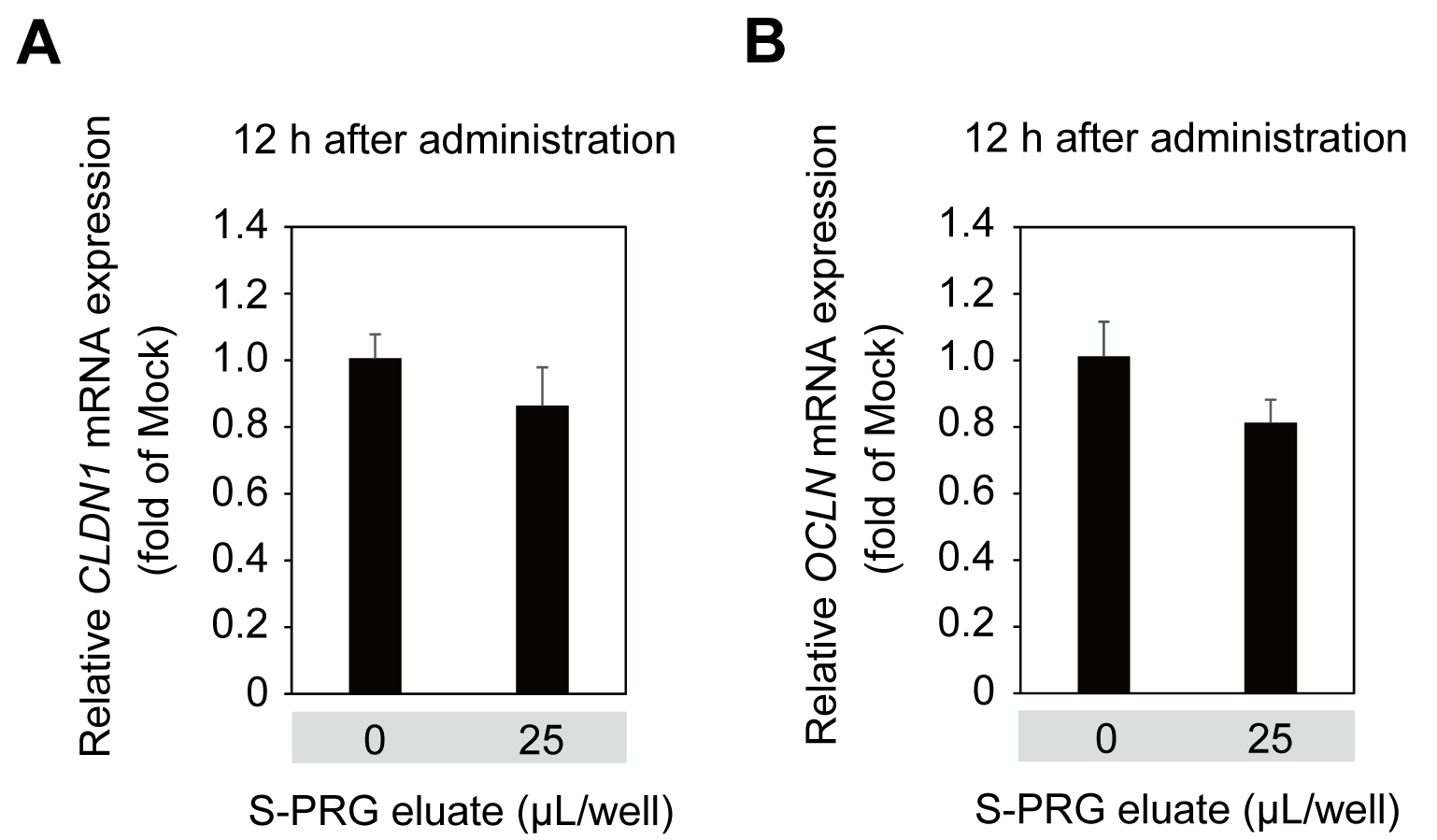

Supplement: S1 Fig — (A, B) Effects of CLDN1 (A) and OCLN (B) mRNA expression in IHGE cells in six-well plates treated with S-PRG eluate for 12 hours. Results are expressed as fold change relative to Mock (no S-PRG eluate) and presented as the mean of five technical replicates. Results shown are representative of two biological replicates. (TIF) [file pone.0271192.s001.tif]

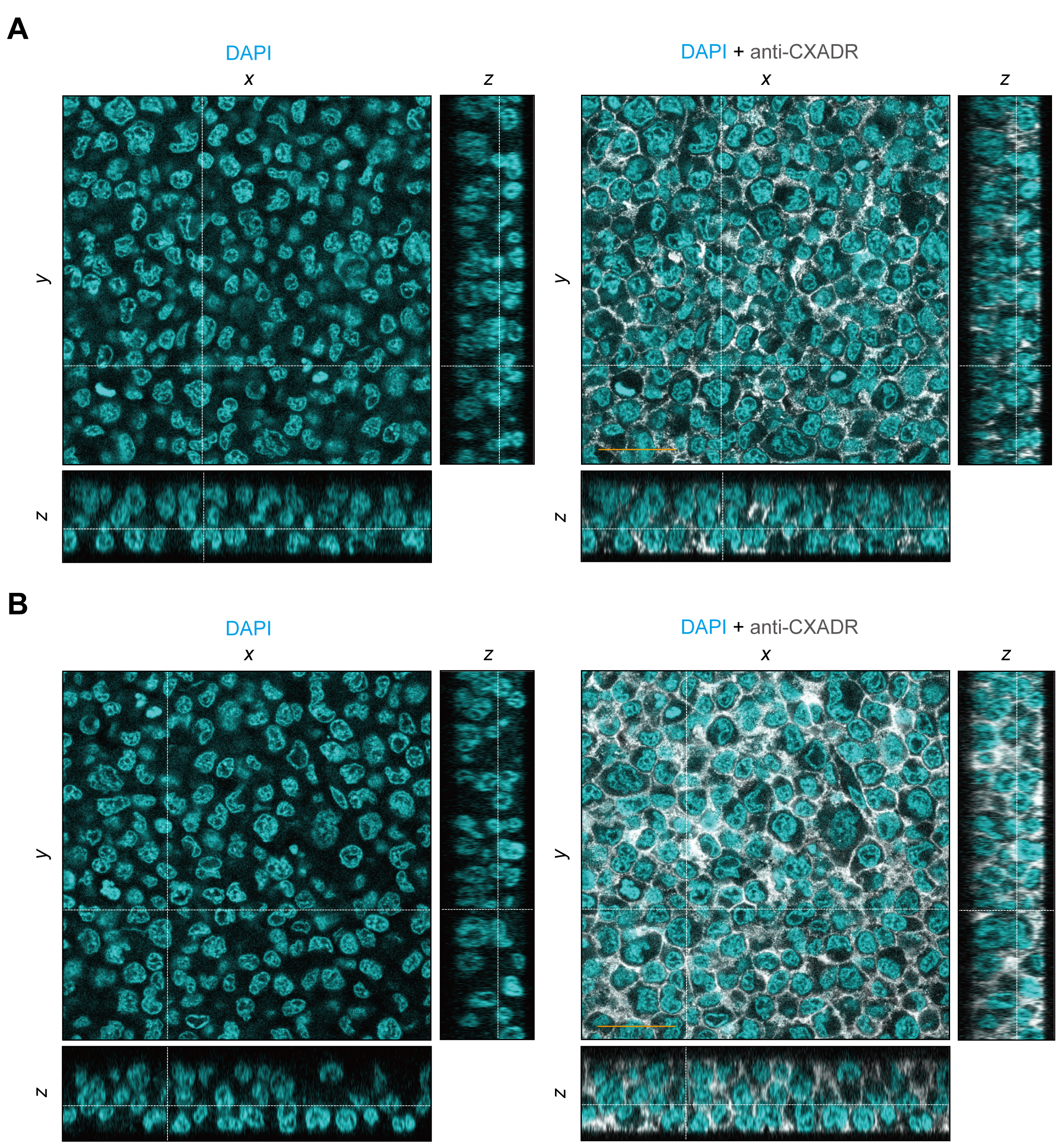

Supplement: S2 Fig — Gingival epithelial tissues on coverslips in 24-well plates were treated with S-PRG eluate for 12 hours, then fixed, stained with DAPI (cyan) and anti-CXADR (gray), and analyzed by confocal microscopy. Scale bars, 30 μm. (TIF) [file pone.0271192.s002.tif]

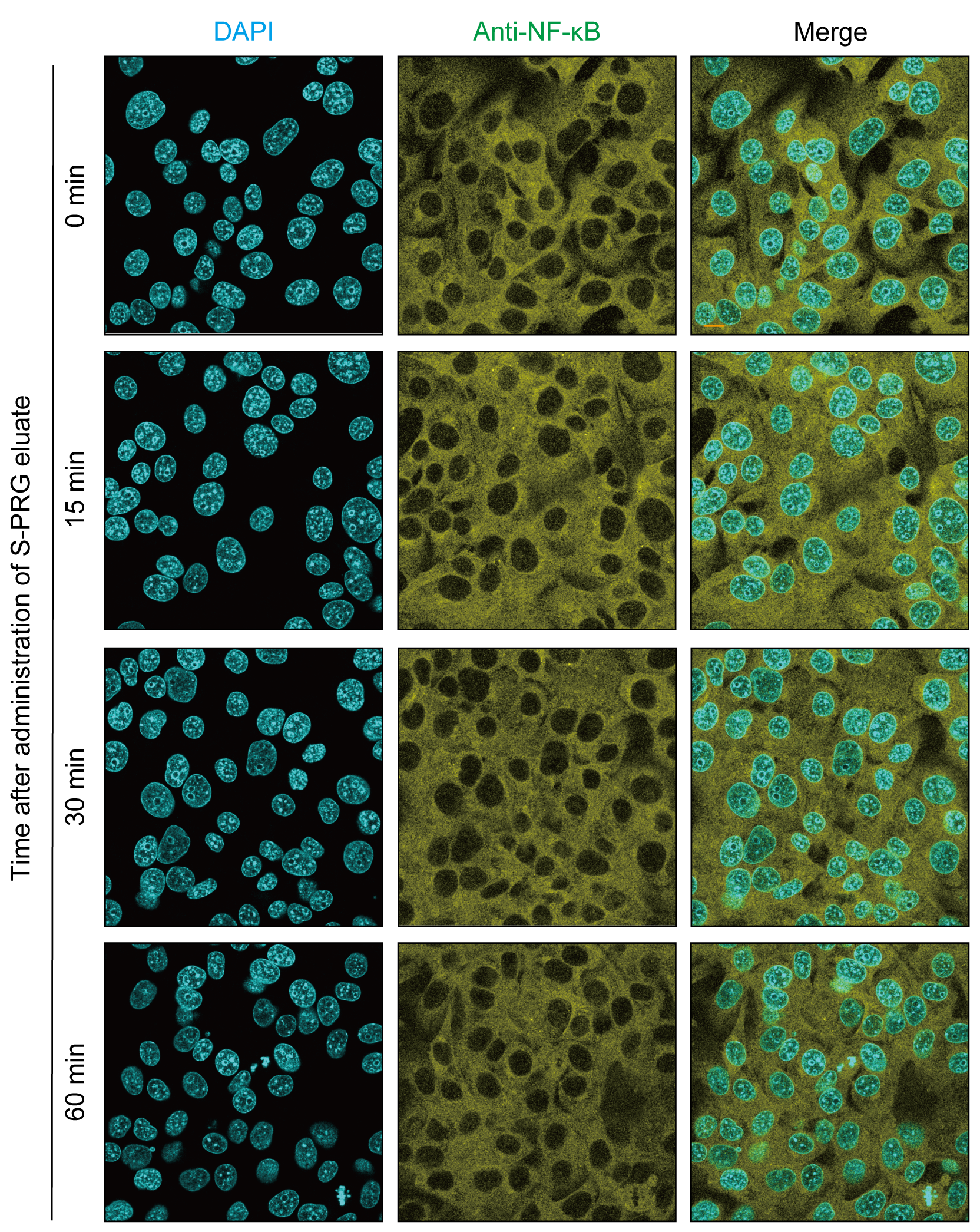

Supplement: S3 Fig — IHGE cells in 12-well plates were treated with 10 μL of S-PRG eluate for the indicated time periods or left unstimulated, then fixed, stained with DAPI (cyan) and anti-NF-κB (yellow), and analyzed by confocal microscopy. Scale bars, 10 μm. Results shown are representative of two biological replicates. (TIF) [file pone.0271192.s003.tif]

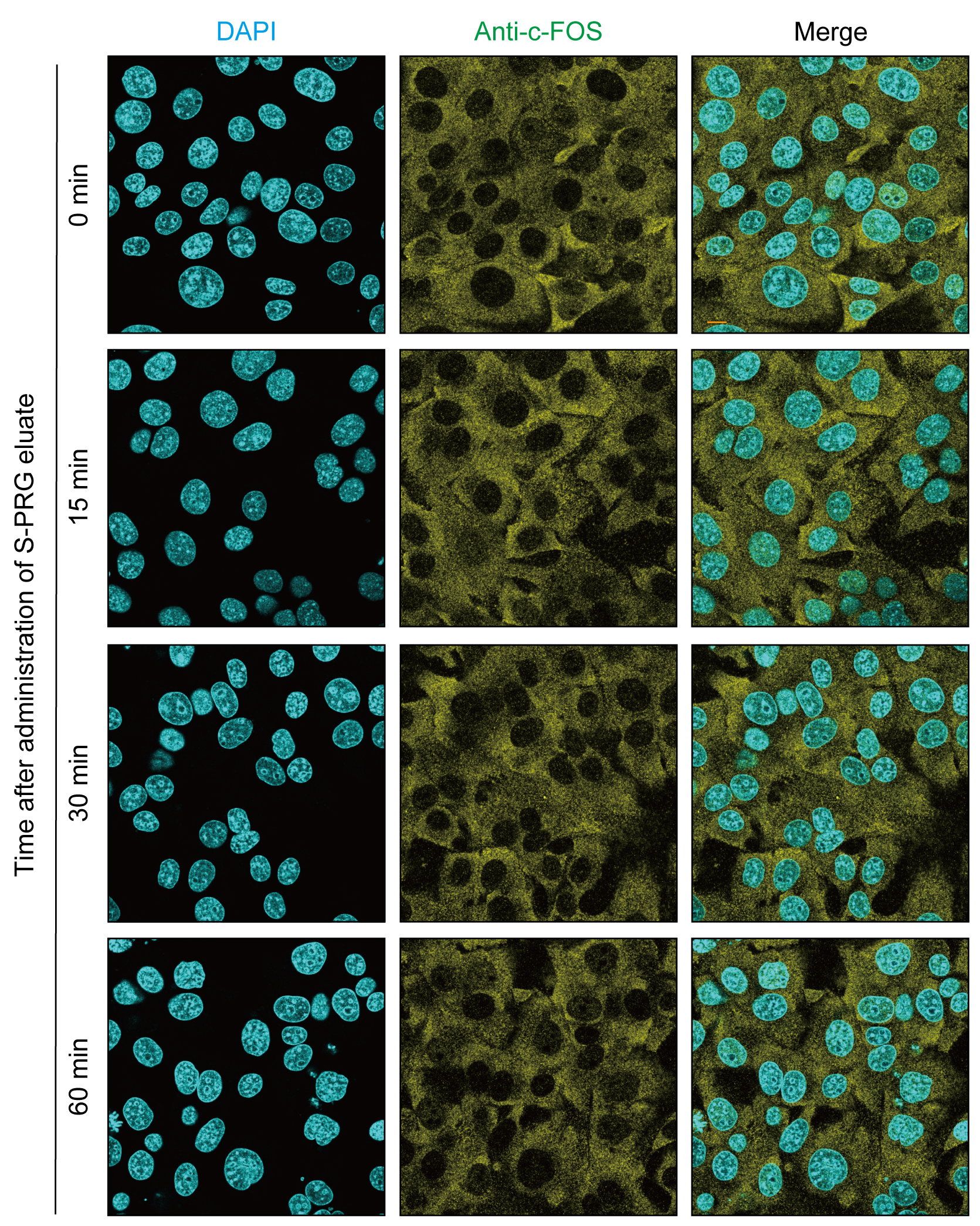

Supplement: S4 Fig — IHGE cells in 12-well plates were treated with 10 μL of S-PRG eluate for the indicated time periods or left unstimulated, then fixed, stained with DAPI (cyan) and anti-c-FOS (yellow), and analyzed by confocal microscopy. Scale bars, 10 μm. Results shown are representative of two biological replicates. (TIF) [file pone.0271192.s004.tif]

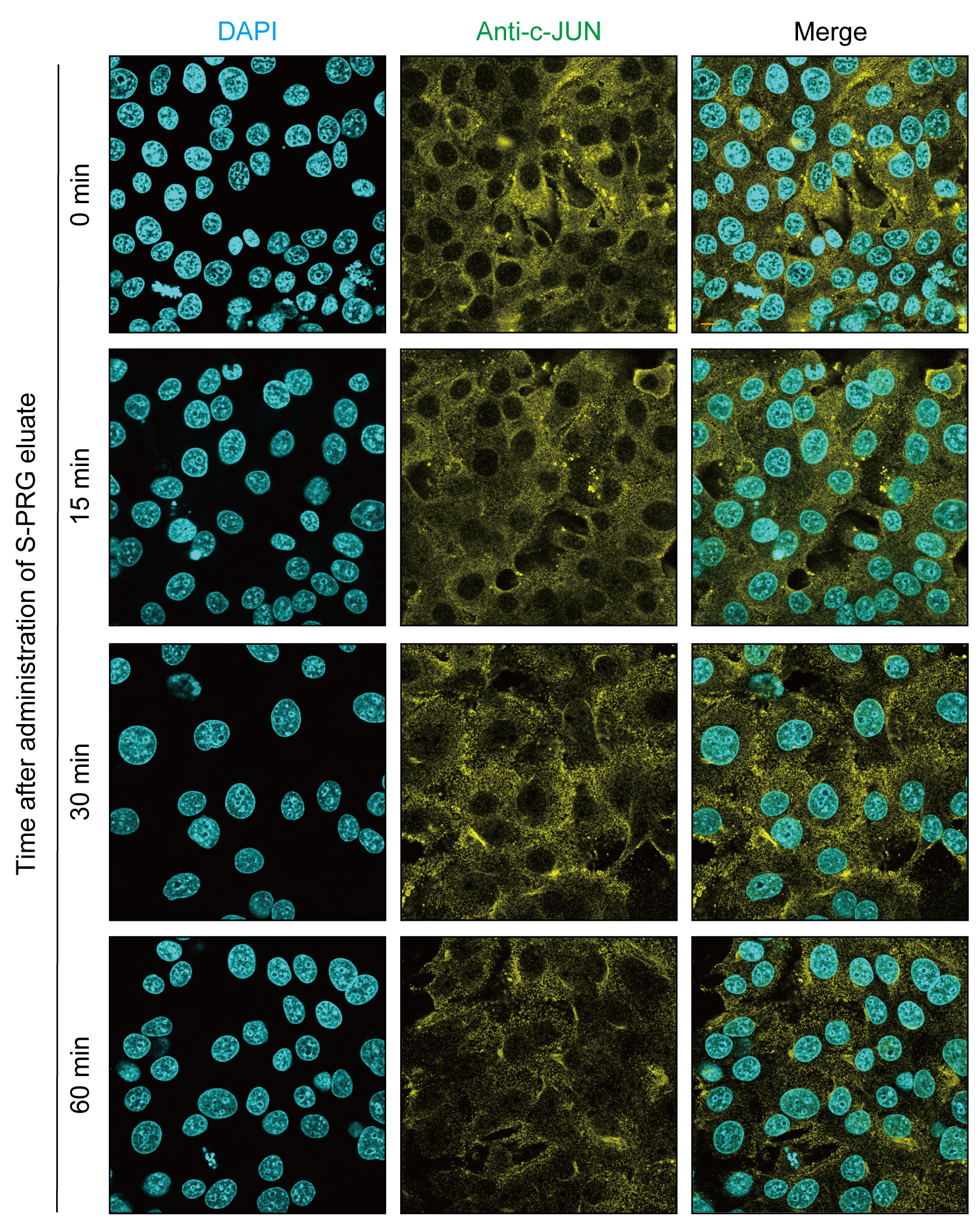

Supplement: S5 Fig — IHGE cells in 12-well plates were treated with 10 μL of S-PRG eluate for the indicated time periods or left unstimulated, then fixed, stained with DAPI (cyan) and anti-c-JUN (yellow), and analyzed by confocal microscopy. Scale bars, 10 μm. Results shown are representative of two biological replicates. (TIF) [file pone.0271192.s005.tif]

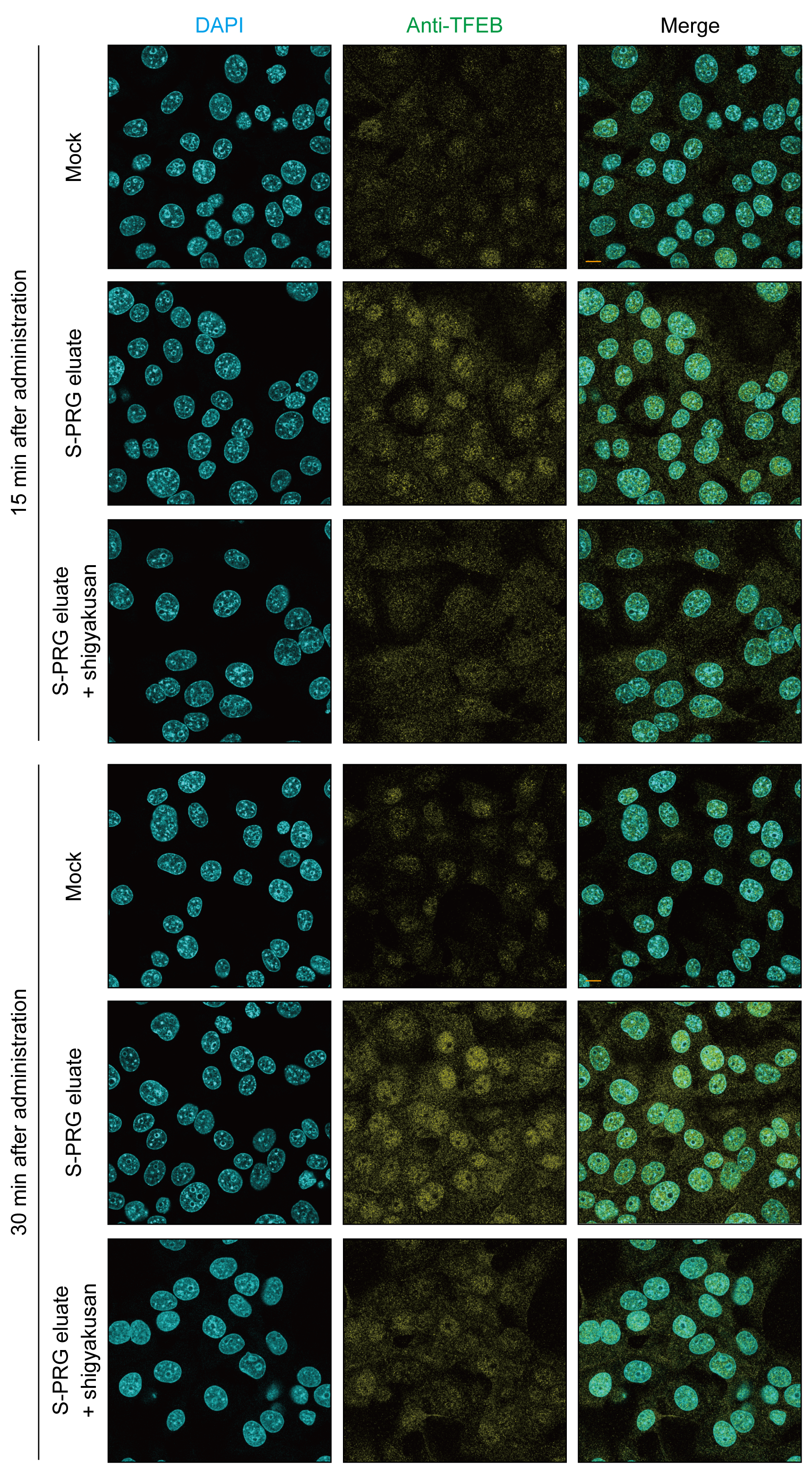

Supplement: S6 Fig — IHGE cells in 12-well plates were treated with 10 μL of S-PRG eluate with or without shigyakusan for the indicted time periods, or left unstimulated, then fixed, stained with DAPI (cyan) and anti-TFEB (yellow), and analyzed by confocal microscopy. Scale bars, 10 μm. Results shown are representative of two biological replicates. (TIF) [file pone.0271192.s006.tif]

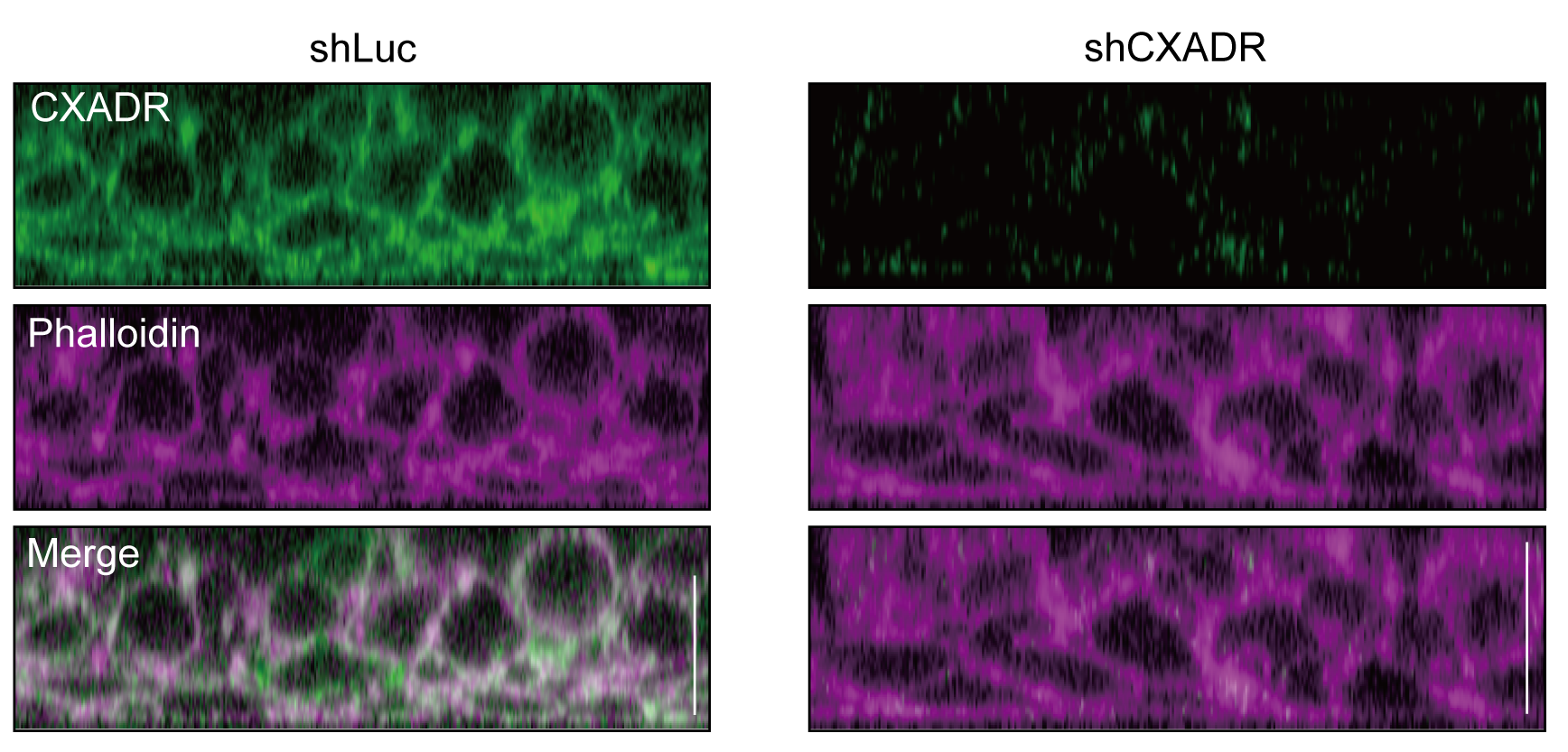

Supplement: S7 Fig — Gingival epithelial tissues stably expressing shLuc or shCXADR on coverslips in 24-well plates were fixed, stained with anti-CXADR (green) and Alexa Fluor 568-conjugated phalloidin (magenta), and analyzed by confocal microscopy. Scale bars, 30 μm. (TIF) [file pone.0271192.s007.tif]

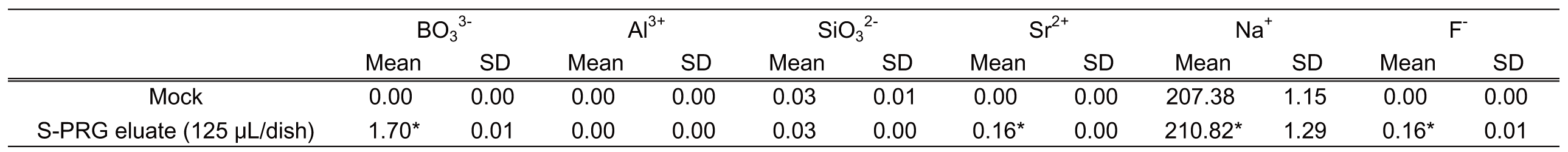

Supplement: S1 Table — IHGE cells in a 10-cm tissue culture dish were treated with S-PRG eluate. After one hour of incubation, cells and culture media were collected and analyzed by ICP-AES for Al3+, BO33-, Na+, SiO32-, and Sr2+, or an ion selective electrode meter for F-. Results are expressed in parts per million (ppm) and presented as the mean of five technical replicates. *p<0.05. (TIF) [file pone.0271192.s009.tif]

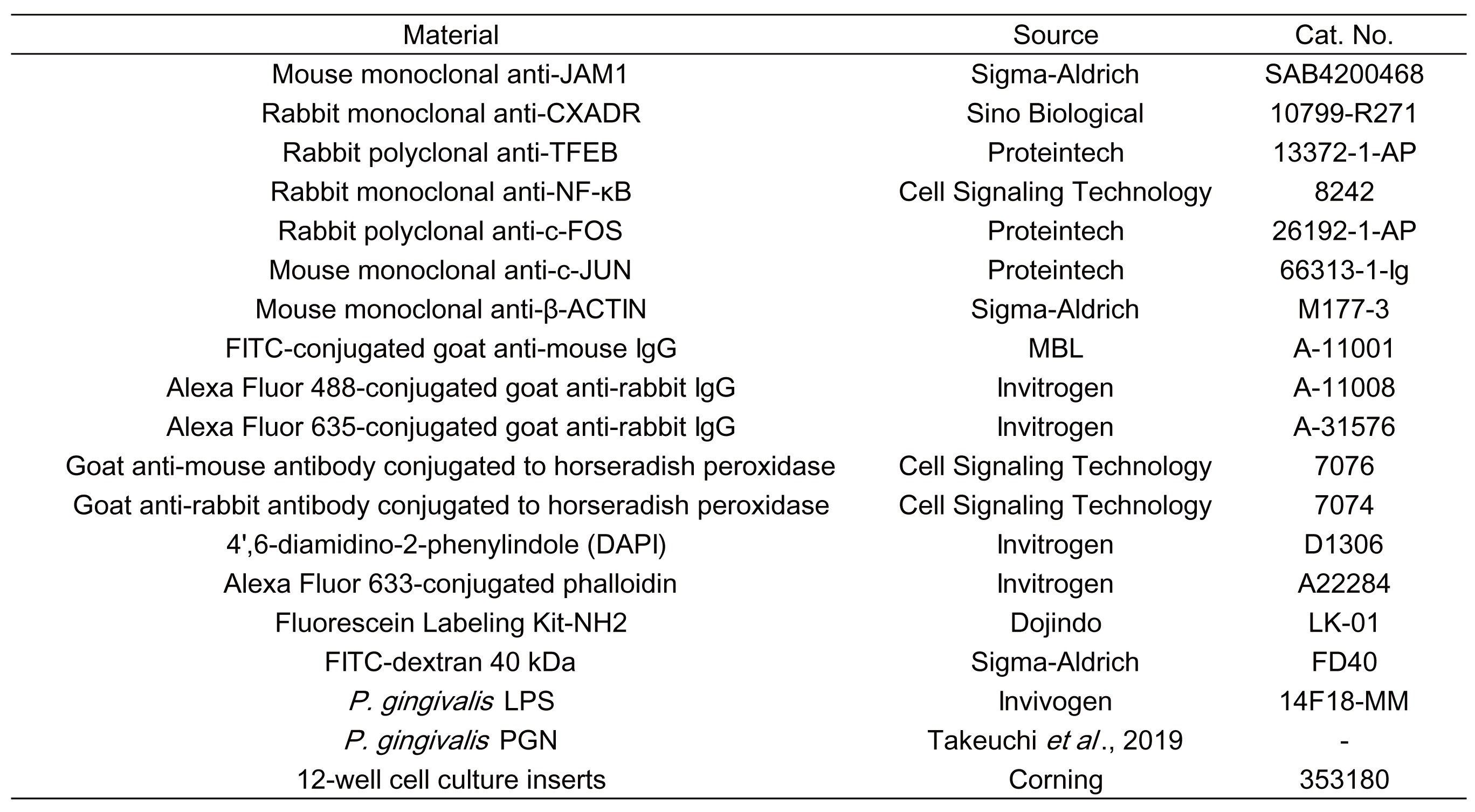

Supplement: S2 Table — (TIF) [file pone.0271192.s010.tif]
