# Supplementary material for: Surface pre-reacted glass-ionomer eluate protects gingival epithelium from penetration by lipopolysaccharides and peptidoglycans via transcription factor EB pathway
Source: PLoS One. 2022 Jul 27;17(7):e0271192. doi: 10.1371/journal.pone.0271192 (PMC9328573; doi:10.1371/journal.pone.0271192)

Figure S8. Immunoblots performed in this study

Figure 1

IB: anti-JAM1

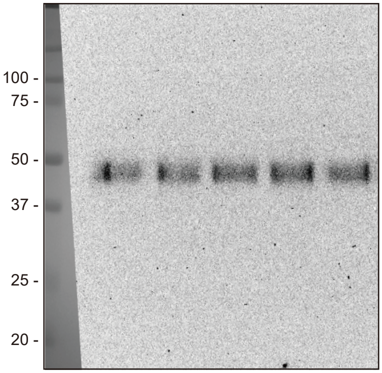

IB: anti-CXADR

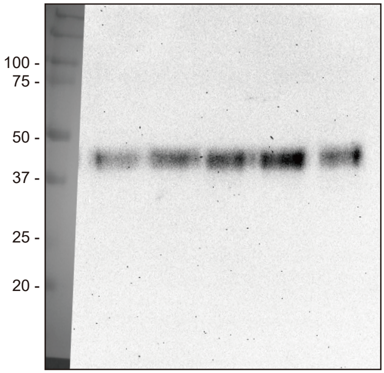

IB: anti-β-ACTIN

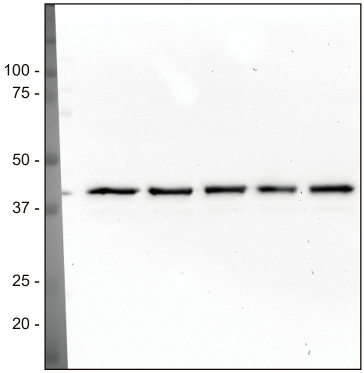

Figure 4

IB: anti-CXADR

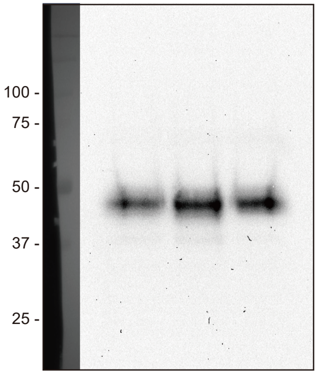

IB: anti-β-ACTIN

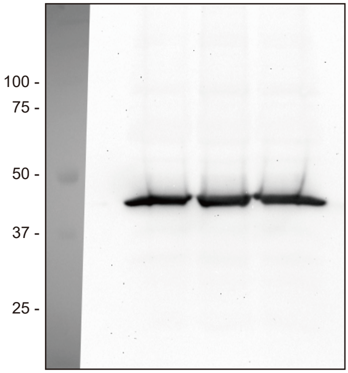

Figure 6

IB: anti-CXADR

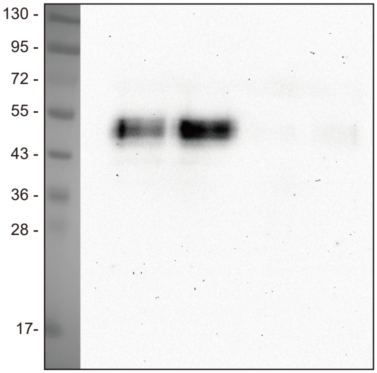

IB: anti-β-ACTIN

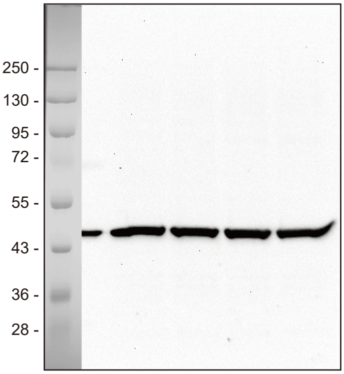

Supplement: S8 Fig — (PDF) [file pone.0271192.s008.pdf]
